# Hotspots and Trends in Research on Early Warning of Infectious Diseases: A Bibliometric Analysis Using CiteSpace

**DOI:** 10.3390/healthcare13111293

**Published:** 2025-05-29

**Authors:** Xue Yang, Hao Wang, Hui Lu

**Affiliations:** Key Laboratory of Public Health Safety and Emergency Prevention and Control Technology of Higher Education Institutions in Jiangsu Province, Department of Social Medicine and Health Education, School of Public Health, Nanjing Medical University, Nanjing 211166, China; yangxxx2000@163.com (X.Y.); wanghao202209@163.com (H.W.)

**Keywords:** CiteSpace, bibliometric analysis, early warning, infectious diseases

## Abstract

**Background:** Emerging and re-emerging infectious diseases (EIDs and Re-EIDs) cause significant economic crises and public health problems worldwide. Epidemics appear to be more frequent, complex, and harder to prevent. Early warning systems can significantly reduce outbreak response times, contributing to better patient outcomes. Improving early warning systems and methods might be one of the most effective responses. This study employs a bibliometric analysis to dissect the global research hotspots and evolutionary trends in the field of infectious disease early warning, with the aim of providing guidance for optimizing public health emergency management strategies. **Methods**: Publications related to the role of early warning systems in detecting and responding to infectious disease outbreaks from 1999 to 2024 were retrieved from the Web of Science Core Collection (WoSCC) database. CiteSpace software was used to analyze the datasets and generate knowledge visualization maps. **Results**: A total of 798 relevant publications are included. The number of annual publications has sharply increased since 2000. The USA produced the highest number of publications and established the most extensive cooperation relationships. The Chinese Center for Disease Control & Prevention was the most productive institution. Drake, John M was the most prolific author, while the World Health Organization and AHMED W were the most cited authors. The top two cited references mainly focused on wastewater surveillance of SARS-CoV-2. The most common keywords were “infectious disease”, “outbreak”, “transmission”, “virus”, and “climate change”. The basic keyword “climate” ranked the first and long duration with the strongest citation burst. “SARS-CoV-2”, “One Health”, “early warning system”, “artificial intelligence (AI)”, and “wastewater-based epidemiology (WBE)” were emerging research foci. **Conclusions**: Over the past two decades, research on early warning of infectious diseases has focused on climate change, influenza, SARS, virus, machine learning, warning signals and systems, artificial intelligence, and so on. Current research hotspots include wastewater-based epidemiology, sewage, One Health, and artificial intelligence, as well as the early warning and monitoring of COVID-19. Research foci in this area have evolved from focusing on climate–disease interactions to pathogen monitoring systems, and ultimately to the “One Health” integrated framework. Our research findings underscore the imperative for public health policymakers to prioritize investments in real-time surveillance infrastructure, particularly wastewater-based epidemiology and AI-driven predictive models, and strengthen interdisciplinary collaboration frameworks under the One Health paradigm. Developing an integrated human–animal–environment monitoring system will serve as a critical development direction for early warning systems for epidemics.

## 1. Introduction

With the acceleration of globalization, interactions between people and animals have become more frequent, significantly increasing the transmission probability of emerging and re-emerging infectious diseases (EIDs and Re-EIDs) [1]. The World Health Organization (WHO)’s 2019 list of the top 10 threats to global health underscores this concern, with four entries directly related to infectious diseases: pandemic influenza, HIV, dengue, and high-threat pathogens such as Ebola [2]. The COVID-19 pandemic has further highlighted that emerging infectious diseases, as a critical and high-priority issue in public health research, continue to pose serious threats to both human physical and mental well-being [3,4].

It is widely acknowledged that effective monitoring and early warning systems are essential to preventing and controlling infectious disease outbreaks at their source [5]. The monitoring and early warning of infectious diseases refer to the long-term, continuous, and systematic collection and analysis of data on the dynamic distribution of infectious diseases within populations and their influencing factors prior to outbreaks or epidemics, aiming to minimize the harm caused by infectious diseases [6]. Critically, such systems are vital for minimizing the spread of EID, particularly in resource-limited settings where delayed responses can lead to catastrophic outcomes. An effective early monitoring and warning system exhibits high sensitivity, universality, and timeliness, enabling healthcare providers to implement rapid containment measures as well as initiate timely patient management strategies [7]. Since the WHO defined the scope and significance of public health monitoring at the 21st World Health Assembly, an increasing number of countries and regions have established monitoring and early warning systems [8].

Bibliometrics employs mathematical and statistical methods to quantitatively analyze large volumes of the literature in a specific research field, revealing key trends and patterns [9]. CiteSpace is a widely used software tool for quantitative analysis and visualization in bibliometrics and knowledge mapping [10]. Despite the increasing recognition that an early warning system is the linchpin of epidemic management, no comprehensive bibliometric study has examined the evolution of infectious disease early warning research. Addressing this gap is essential for optimizing global surveillance strategies and guiding the construction of future public health emergencies. Thus, we perform a bibliometric analysis of infectious disease early warning research from 1999 to 2024 using CiteSpace. Our study not only maps the current landscape of this critical field, but also identifies future directions to strengthen pandemic preparedness. These findings provide policymakers and public health practitioners with insights to improve surveillance strategies, thereby alleviating the global burden of infectious diseases. Specifically, we address the following research questions:

(1) Who are the major contributors (disciplines, authors, institutions, and countries) to the field?
(2) What are the hotspots and frontiers in the field?

## 2. Materials and Methods

### 2.1. Data Sources

We derived the literature under study from the Science Citation Index Expanded (SCI-Expanded) of the Web of Science database. WoSCC prioritizes peer-reviewed journals in core disciplines such as epidemiology and public health, ensuring access to high-quality, relevant literature for infectious disease early warning research [11]. Moreover, WoSCC demonstrates exceptional compatibility with CiteSpace’s co-citation algorithms, as its data can be directly recognized and processed using CiteSpace [12], safeguarding the reliability and consistency of the analytical results.

The search was conducted based on the topic (TS), and the search term was set to TS = “infectious disease” and TS = “warning*”. The type of literature was limited to “article” or “review article” and the language was limited to English. The temporal scope of our literature search spanned from the database’s inception to the retrieval date (5 September 2024). This temporal scope has been carefully designed to achieve dual objectives: it ensures systematic literature retrieval through comprehensive coverage of the complete historical dataset while simultaneously maximizing the inclusion of cutting-edge research outputs, thereby fully capturing the most current advancements in the field.

Following the search protocol, we initially identified 915 potentially relevant articles. After removing duplicate records, 906 unique publications remained for preliminary evaluation. To ensure data quality and relevance, we systematically reviewed all abstracts, excluding studies that did not involve human subjects or focus on infectious diseases. Finally, we exported 798 documents in plain text format, including full records and cited references, for subsequent bibliometric analysis.

### 2.2. Research Method

For literature-based research, understanding a field’s landscape, hotspots, and trends is crucial. With the growth of data-driven approaches, mining research hotspots and trends from the existing literature using scientific methods have become increasingly common. CiteSpace, a bibliometric visualization tool, is widely used for systematic literature analysis and review writing. Previous investigations have employed CiteSpace to visualize the evolutionary trajectories of diverse healthcare disciplines. It can be used to construct visual knowledge frameworks by analyzing keyword co-occurrence networks, document co-citation clusters, and emergent theme detection. These visualizations are structured around nodes and edges: nodes signify research entities such as keywords, authors, and journals, with their size proportional to centrality and occurrence frequency; edges represent semantic or citation relationships, where thickness and density indicate the strength of interconnectedness. Notably, CiteSpace’s burst detection algorithm identifies terms experiencing sudden surges in usage, enabling the tracking of emerging trends or declining topics. By mapping these dynamic shifts, the software facilitates the identification of research frontiers and knowledge gaps within academic landscapes. Such analytical capabilities have proven to be instrumental in synthesizing interdisciplinary research and guiding future inquiries.

In this study, we used CiteSpace 6.3.R3 (64 bit) Advanced software to perform the analysis, and the parameters were set as follows:

(1) Timespan: 1999–2024 (timespan from first publication to search termination date);
(2) Time slice: 1 year;
(3) Node type = country/institution/author/journal/keyword/cited reference;
(4) Threshold selection criteria = the top 25 results for each time slice (balancing computational feasibility with network representativeness, capturing the vast majority of important nodes while minimizing noise);
(5) Other parameters were kept at default settings.

## 3. Results

### 3.1. Basic Statistical Analysis

**Figure 1:**
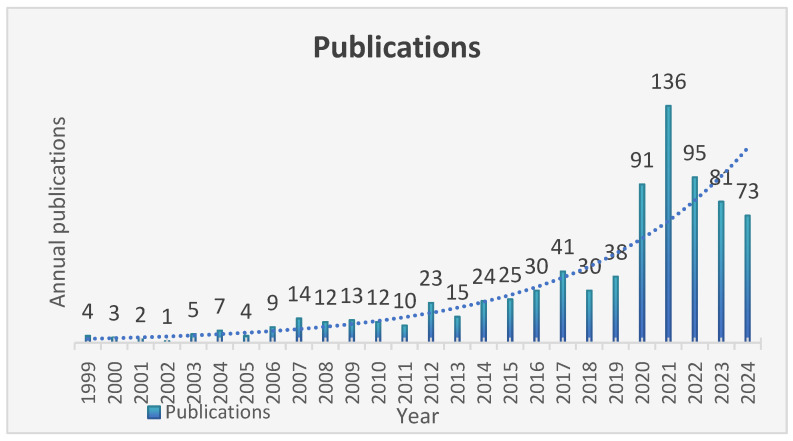
The annual number of publications from 1999 to 2024.

Figure 1 depicts the annual publication trend of 798 studies from 1999 to 2024, showing an overall fluctuating upward trend. Between 1999 and 2011, publications on infectious disease early warning systems remained relatively scarce, with an average of 7.4 per year. From 2012 to 2019, scholarly attention toward early warning systems for EIDs gradually increased, with publication numbers showing a steady upward trend. A significant surge occurred after 2020, peaking in 2021 with 136 publications before gradually declining. Figure 1 directly demonstrates that infectious disease early warning technologies and systems have emerged as a prominent research focus in recent years.

### 3.2. Co-Occurrence Analysis of Countries and Institutions

**Figure 2:**
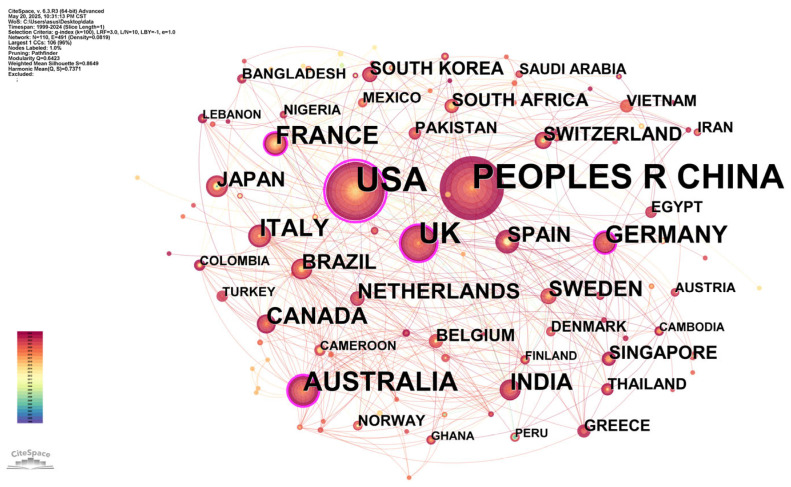
Country collaboration map. Visualization created using CiteSpace software. Nodes represent individual countries participating in research collaboration, where node size corresponds to the frequency of each country’s research output. Connecting lines signify collaborative relationships between countries, with edge thickness reflecting the intensity of cooperative engagement. The legend in the lower left corner shows the mapping of colors to time. The bottom corresponds to the year 1999, the top corresponds to the year 2024, and the time gradually moves backwards from cooler to warmer colors.

The geographical distribution analysis reveals national contributions to this research field. Figure 2 demonstrates the robust collaborative relationships among countries contributing publications in this research field. In total, 113 countries released 798 records in the past 20 years. The United States and China are the most productive nations, contributing 235 (29.4%) and 230 (28.8%) publications, respectively, establishing their global leadership in this field. Notably, the United States demonstrated the highest centrality score (0.48), indicating its pivotal role in international research collaborations. The network visualization reveals distinct geographical disparities in research productivity. Developed nations—particularly the United States, United Kingdom, Spain, Australia, and Germany—demonstrate clear dominance in infectious disease early warning research. This stands in contrast to the overall limited output from developing economies. However, certain Asian developing countries show notable research activity, with Vietnam, Thailand, China, and India all contributing meaningful publications. Notably, China and India represent the only developing nations ranking among the top ten most prolific countries in terms of publication volume. While China’s publication volume rivals that of the United States (235 vs. 230 articles), its lower centrality score (0.05 vs. 0.48) indicates comparatively limited international collaboration.

Table 1 illustrates the Top 10 institutions in terms of highest centrality. The top 10 publishing institutions comprise leading American universities, such as Harvard University and the University of California System, British, Australian, and French academic institutions, the World Health Organization, and specialized Chinese medical research centers. The institutional rankings mirror broader national research trends, corresponding directly to the prominent roles played by the United States, United Kingdom, France, Australia, and China in global infectious disease surveillance research. This alignment demonstrates how national research priorities and investments translate into institutional leadership positions within the field.

Notably, the WHO demonstrated the highest centrality (0.22), indicating its predominant influence within the collaborative network. Among the top 10 publishing institutions, a clear dichotomy emerges: while American universities, British universities, and other Western academic institutions dominate the list, China’s contributions are primarily driven by specialized research organizations like the Chinese Center for Disease Control & Prevention and the Chinese Academy of Sciences. This distribution reveals two critical observations: Western universities serve as primary research engines, likely due to greater resource allocation for scholarly work within their academic structures, and China’s specialized research institutions achieve comparable output through targeted resource concentration in dedicated public health research programs. The collaboration network analysis reveals strong collaboration networks among American and European institutions, but limited cooperation between developed and developing nations, particularly across continental divides (America–Europe vs. Asia–Africa). This disparity suggests an urgent need for more equitable global partnerships to bridge the research capacity gap.

### 3.3. Analysis of Authors and Co-Cited Authors

**Figure 3:**
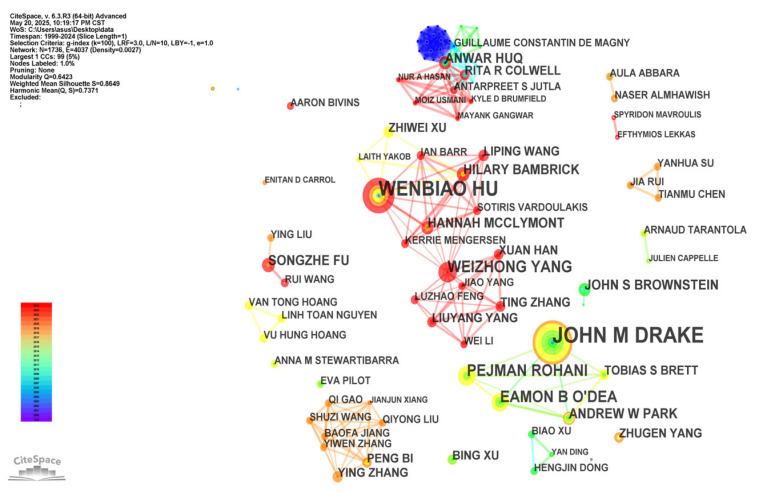
Co-occurrence map of authors. The visual representation of co-authorship networks was generated using CiteSpace. Each node corresponds to an individual author, and edges signify co-authorship relationships. The number of shared publications weights edge thickness, while node size is scaled according to each author’s prominence in the network. The legend in the lower left corner shows the mapping of colors to time. The bottom corresponds to the year 1999, the top corresponds to the year 2024, and the time gradually moves backwards from cooler to warmer colors.

Figure 3 presents the author collaboration network, where node dimensions reflect individual publication productivity, and the thickness of connecting edges denotes the strength of the collaborative relationships The top 10 most prolific authors collectively published 65 articles, making substantial contributions to the field. Drake, John M emerged as the most prolific author (16 articles), followed by Hu Wenbiao (10), Yang Weizhong (7), Brownstein, John S (6), and Colwell, Rita R (5). Notably, these high-output authors exhibit paradoxically low network centrality (all ≤ 0.05) and minimal direct collaboration, forming no interconnected clusters, despite their individual productivity.

**Figure 4:**
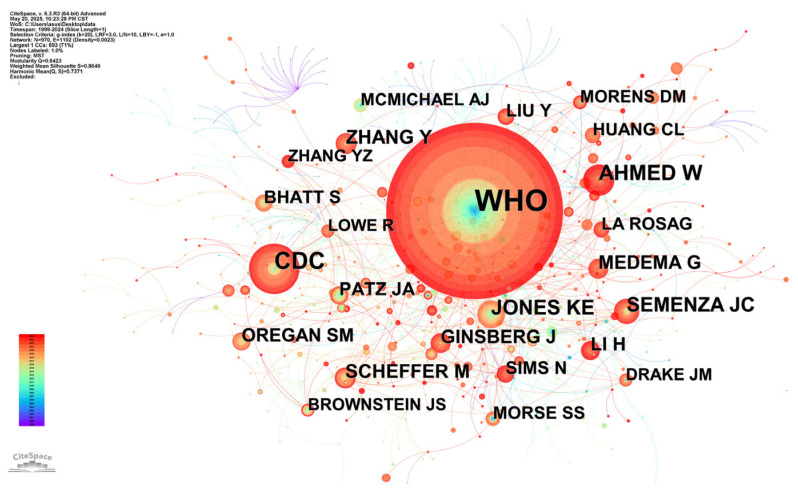
Co-occurrence map of co-cited authors. The visual representation of co-cited author networks was generated using CiteSpace. Each node corresponds to an individual author, and edges signify co-cited relationships. The number of times two authors are co-cited together weights edge thickness, while node size is scaled according to each author’s citation prominence in the network, such as total citation count. This visualization allows for the identification of clusters of highly co-cited authors and the strength of connections between them, highlighting key figures and collaborative relationships within the research field. The legend in the lower left corner shows the mapping of colors to time. The bottom corresponds to the year 1999, the top corresponds to the year 2024, and the time gradually moves backwards from cooler to warmer colors. In the figure, WHO stands for World Health Organization.

An author co-citation analysis identified frequently co-cited authors within published research, enabling the recognition of influential scholars in a specific field. Figure 4 displays this network, where node size corresponds to citation frequency. Analysis revealed the WHO to be the most cited author (198 citations), followed by AHMED W (36), JONES KE (32), and SEMENZA JC (29). The WHO’s prominent citation reflects its global leadership in public health initiatives. The network topology exhibits a distinct radial structure with the WHO at the center and limited interconnection among peripheral authors, suggesting that, while these scholars contribute significantly to specialized subdomains, their work develops along relatively independent trajectories. The uniformly low centrality among top-cited authors (all scores = 0) indicates minimal co-citation relationships between these influential sources, with collaboration occurring primarily at institutional rather than individual researcher levels.

### 3.4. Dual-Map Overlay Analysis of Journals

By analyzing this journal-level overlay, we identified patterns of cross-disciplinary knowledge diffusion within the field of infectious disease early warning systems. The visualization highlights how research in this domain draws upon and contributes to diverse disciplinary areas, offering insights into the interdisciplinary nature of infectious disease surveillance and prediction.

**Figure 5:**
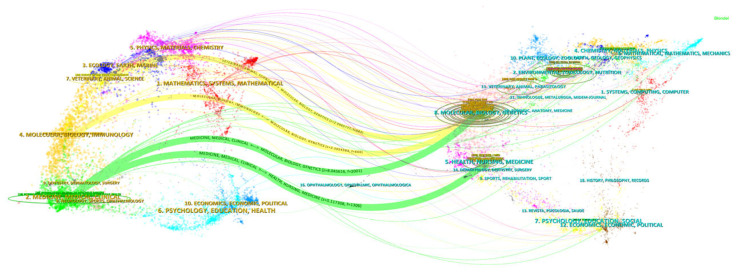
Overlay of dual images of journals of early warning of infectious diseases. A dual-map overlay visualization was generated by CiteSpace to illustrate the interdisciplinary knowledge transfer between citing journals and cited journals. The left map represents citing journal domains, while the right map shows cited journal domains. By juxtaposing these two maps in an overlay, researchers can comprehensively analyze how knowledge is transferred across different journal domains. This includes identifying key bridging journals, understanding the evolution of research topics, and detecting emerging.

In Figure 5, the left map represents citing journal domains (“#1 Mathematics/Systems/Mathematical”, “#2Medicine/Mmedical/Clinical”, “#3Ecology/Earth/Marine”, “#4Molecular/Biology/Immunology”, “#5Physics/Materials/Chemistry”, “#6Psychology/Education/Health”, “#7Veterinary/Animal/Science”, “#8Neurology/Sports/Ophthalmology”, “#9Dentistry/Dermatology/Surgery”, “#10Economics/Economic/Political”), (“#1Systems/Computing/Computer”, “#2Environmental/Toxicology/Nutrition”, “#4Chemistry/Materials/Physics”, “#5Health/Nursing/Medicine”, “#6Mathematical/Mathematics/Mechanics”, “#7Psychology/Education/Social”, “#8Molecular/Biology/Genetics”, “#9Sports/Rehabilitation/Sport”, “#10Plant/Ecology/Zoologyth/Geology/Geophysics”). The former can be seen as the field application of the early warning of infectious diseases, and the latter can be seen as the research basis of the early warning of infectious diseases. The citation trajectories between domains indicate knowledge flow patterns, revealing that the discipline transformation still mainly remained in molecular biology, medicine, and research, primarily transitions from molecular biology foundations to clinical applications in infectious disease early warning systems.

### 3.5. Analysis of Keywords

#### 3.5.1. Analysis of Keyword Co-Occurrence

We used a keyword co-occurrence analysis to examine the frequency of keyword pairs appearing together, revealing research hotspots and trends. Table 2 presents high-frequency keywords ranked by the frequency of occurrence and centrality. Keywords related to the early warning of infectious diseases mainly include infectious diseases, transmission, virus, outbreak, climate change, disease, public health, surveillance, infection, and epidemiology. Notably, the keywords “climate change”, “diseases”, “outbreak”, “public health”, and “infectious diseases” appear in both the top ten centrality and frequency rankings, highlighting their critical importance in this research field. The prominence of “climate change” as a key theme underscores the academic recognition of environmental drivers in disease transmission dynamics, while terms like “surveillance” and “epidemiology” reflect the field’s methodological priorities. The co-occurrence patterns of these keywords demonstrate a significant interdisciplinary convergence, particularly between climate science and public health, providing multidimensional perspectives for infectious disease early warning research.

**Figure 6:**
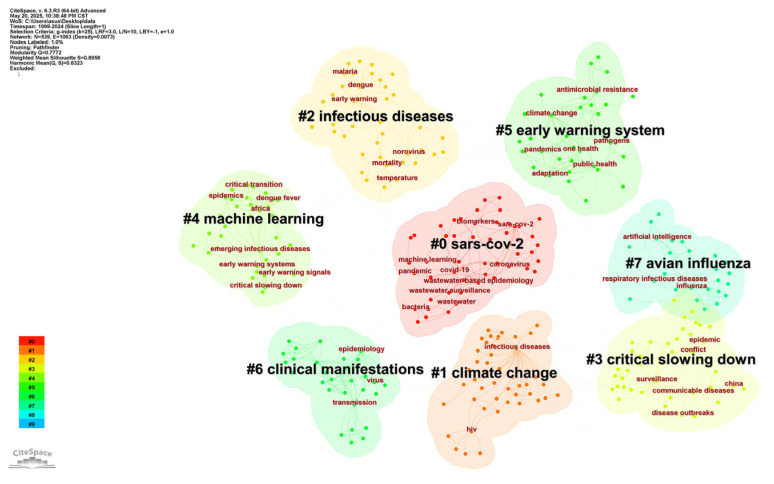
Cluster map of keywords. This visual map was generated using CiteSpace to display the distribution of keyword clusters. In the map, regions of different colors correspond to different keyword clusters, respectively. These clusters are identified through modular algorithms, aggregating related keywords at the semantic level. Within each cluster, keywords that are centrally located and have relatively large node sizes constitute the core theme of that cluster. Other specific keywords surrounding the core keywords are closely associated with the theme keywords, refining and expanding the research scope of this theme.

The cluster analysis of keyword co-occurrences (Figure 6) identified several research themes. The clusters tagged as “#0 SARS-CoV-2” ranked first place, followed by “#1 Climate change”, “#2 Infectious diseases”, “#3 Critical slowing down”, “#4 Machine learning”, “#5 Earlywarming system”, “#6 Clinical manifestations”, and “#7 Avian influenza”.

The most important research cluster, “#0 SARS-CoV-2”, dominates the research landscape, reflecting the scientific community’s great interest in the COVID-19 pandemic. This research cluster includes wastewater surveillance, genome sequencing, and transmission modeling studies for SARS-CoV-2, showing how a novel pathogen rapidly reshaped research priorities. The closely following “#1 Climate Change” cluster highlights the growing recognition of environmental factors in the emergence and spread of disease, including research on vector-borne diseases, zoonotic spillover events, and the development of climate-informed early warning systems. “#4 Machine Learning” demonstrates the field’s increasing reliance on artificial intelligence for pattern recognition and predictive modeling. Clinical perspectives emerged in the “#6 Clinical manifestations” group, emphasizing the continuing importance of symptom-based surveillance and the need to correlate population-based warnings with clinical outcomes.

The coexistence of these research clusters demonstrates the multifaceted nature of contemporary infectious disease early warning systems, in which four synergistic areas collectively improve surveillance capabilities: (1) pathogen-specific investigations (e.g., SARS-CoV-2 genome surveillance), (2) environmental determinants (especially the impact of climate change on vector ecology), (3) technological innovations (e.g., machine learning), and (4) clinical translation (real-time syndromic surveillance integration).

**Figure 7:**
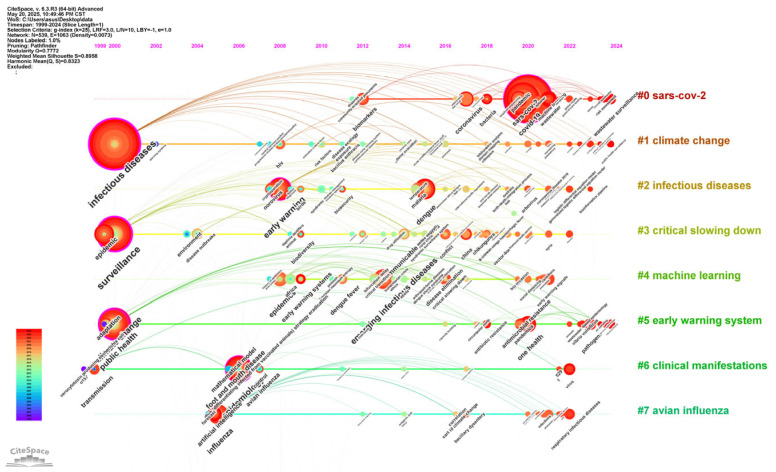
Timeline view of keywords. Visualization generated using CiteSpace depicting the evolution of keyword co-occurrence networks over time. Nodes represent keywords, sized by total occurrence frequency. The position of a node on the timeline corresponds to the time when the keyword emerged, which can reflect the keyword’s activity level in different time periods (from left to right represents time from early to late). Lines indicate co-occurrence relationships, with thickness proportional to co-occurrence strength. The legend in the lower left corner shows the mapping of colors to time. The bottom corresponds to the year 1999, the top corresponds to the year 2024, and the time gradually moves backwards from cooler to warmer colors. The phrases in the figure are clusters of semantically related keywords identified through modularity optimization.

Based on the clustering results, we generated a timeline visualization to track the temporal evolution of these keywords (Figure 7). The horizontal axis represents the publication years, while the vertical axis displays different clusters. Each node corresponds to a keyword, with size indicating the frequency of occurrence. The timeline reveals a continuous increase in keywords related to infectious diseases (1999–2024), with a sharp surge in COVID-19 (2020–2022), reflecting pandemic-driven research priorities. Prior to 2020, predominant keywords include “surveillance”, “infectious diseases”, “norovirus”, “malarias”, “environment”, “influenza”, and “climate”, indicating that the development of disease surveillance technologies, the mechanisms of transmission of specific pathogens, and the impact of environmental and climatic factors on infectious diseases are the focus of early attention. During the peak of the pandemic in 2020–2022, “SARS-CoV-2”, “COVID-19”, and “pandemic” emerged as dominant keywords, highlighting the urgent global focus on understanding the novel coronavirus, its disease manifestations, and pandemic-scale strategies. Recent keywords like “One Health” and “wastewater surveillance” signaled a shift towards an interdisciplinary ecosystem-based approach.

Synthesizing the timeline, keyword evolution reflects a transition from reactive disease control to proactive multi-sectoral surveillance. This visualization provides a historical context for understanding research priorities and identifies emerging frontiers in infectious disease early warning systems.

#### 3.5.2. Analysis of Keyword Bursts

**Figure 8:**
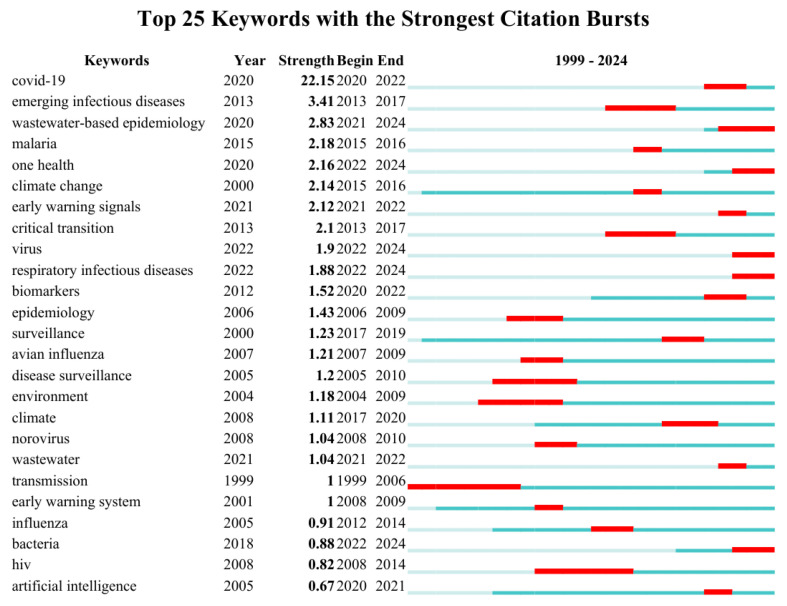
A list of the top 25 keywords exhibits the strongest citation bursts between 1999 and 2024, as determined by CiteSpace. Each row shows the keyword, its burst strength, and the beginning/end years of the citation surge. The blue line indicates the study period, while red segments denote burst durations, characterized by both intensity and temporal span.

Analyzing keyword burst visualizations can help identify emerging research frontiers and trace the developmental trajectory of the research area. Figure 8 presents the top 25 keywords exhibiting the strongest citation bursts during the period from 1999 to 2024. The analysis of keyword bursts reveals three distinct phases in the evolution of infectious disease early warning research, each characterized by specific thematic focuses and reflecting broader shifts in public health paradigms: (1) 2004–2016: “climate change”, “infectious diseases”, “environment”; (2) 2016–2022: “COVID-19”, “influenza”, “surveillance”; (3) 2022–2024: “One Health”, “Wastewater-based epidemiology”, “system”.

The keywords in the earliest phase (2004–2016) were “climate change”, “infectious diseases”, and “environment”. Research during this period primarily focused on employing epidemiological approaches to investigate environmental determinants of disease transmission, particularly the associations between pathogen spread and ecological factors. Studies during this period provided an important foundation for understanding the environmental drivers of disease emergence, while also revealing the limitations of climate-only approaches to disease prediction. The subsequent period (2016–2022) witnessed a remarkable shift in infectious disease early warning, with site-specific “COVID-19” and pathogen-specific “influenza”. These phased keyword occurrences document the field’s conceptual evolution from investigating disease–environment linkages to implementing pathogen-specific surveillance systems, reflecting a paradigm shift toward outbreak-driven real-time research methodologies. The most recent phase (2022–2024) shows the maturation of the field towards systems integration, as evidenced by the burst of the keywords “Wastewater-based epidemiology”, “One Health”, and “system”. The transition from pathogen-specific to systems-level approaches suggests that the field has learnt the lessons of previous phases, particularly the need for interdisciplinary collaboration and the value of computational approaches in managing complex disease systems. The emergence of “One Health” as an emergent keyword is particularly important, formally recognizing the interconnectedness of human, animal, and environmental health in disease surveillance—a concept that evolved directly from earlier climate-focused research.

Using a systematic analysis of keyword burst maps and timeline visualization, we can delineate the evolutionary trajectory of infectious disease early warning research. Research foci in this area have evolved from focusing on climate–disease interactions to pathogen monitoring systems and ultimately to the “One Health” integrated framework.

### 3.6. Analysis of Cited References

Table 3 lists the top 10 most frequently co-cited references, including four papers cited more than 15 times and all papers cited at least 10 times. When two publications are cited jointly by a third citing publication, this is defined as a co-citation relationship [13]. In general, the number of times a publication is cited represents its importance in a certain field. Thus, by analyzing the most frequently cited publications, we can grasp the hotspots in this field.

The most cited document is the one published by Ahmed in 2020, with 25 citations. Sims N’s publication in 2020 ranks second in 2020, garnering 19 citations, while Huang CL’s publication in 2020 secures the third position with 17 citations. Notably, these highly cited references predominantly originate from prestigious journals, such as *Lancet* and *Nature*, and comprise seven epidemiological studies, one clinical trial, and two review articles. Six epidemiological studies focus on SARS-CoV-2. The top two references primarily address wastewater surveillance of SARS-CoV-2. Ahmed W et al. [14] pioneered the application of RT-qPCR to detect SARS-CoV-2 in Australian wastewater systems, thereby validating wastewater-based epidemiology as a viable approach for community-level COVID-19 surveillance. The wastewater monitoring approach is referred to as wastewater-based epidemiology. This methodology, grounded in the extraction, detection, and subsequent analysis of chemical and biological markers, has since been instrumental in tracking the prevalence of various infectious pathogens [15,16]. Sims N et al. [17] provided a comprehensive review of current infectious disease surveillance frameworks, emphasizing the emerging role of WBE as a complementary tool for public health monitoring and its technological advancements. Specifically, the authors highlight the need for standardized protocols and longitudinal data collection to maximize WBE’s utility in infectious disease management. Huang CL et al. [18], Zhou F et al. [19], and Guan W et al. [20] reported the clinical characteristics of SARS-CoV-2 in China, respectively. These papers contributed significant clinical data on SARS-CoV-2, as evidenced by the fact that they were cited in several publications. Brett TS et al. [21] provide a theoretical basis for the development of methods to anticipate disease emergence. In summary, these ten highly cited references show the clinical characteristics of SARS-CoV-2, the application and development of wastewater-based epidemiology, and the exploration of establishing infectious disease prediction models.

**Table 3:** Top 10 cited references ranked according to frequency.

Rank	Frequency	Year	Article Title	Journal Title
1	25	2020	First confirmed detection of SARS-CoV-2 in untreated wastewater in Australia: A proof of concept for the wastewater surveillance of COVID-19 in the community [14]	*Science of the Total Environment*
2	19	2020	Future perspectives of wastewater-based epidemiology: Monitoring infectious disease spread and resistance to the community level [17]	*Environment International*
3	17	2020	Clinical features of patients infected with 2019 novel coronavirus in Wuhan, China [18]	*The Lancet*
4	17	2020	Presence of SARS-Coronavirus-2 RNA in Sewage and Correlation with Reported COVID-19 Prevalence in the Early Stage of the Epidemic in The Netherlands [22]	*Environmental Science & Technology Letters*
5	14	2020	Clinical course and risk factors for mortality of adult inpatients with COVID-19 in Wuhan, China: a retrospective cohort study [23]	*The Lancet*
6	14	2020	Clinical Characteristics of Coronavirus Disease 2019 in China [20]	*The New England Journal of Medicine*
7	14	2020	A pneumonia outbreak associated with a new coronavirus of probable bat origin [19]	*Nature*
8	10	2020	The effect of human mobility and control measures on the COVID-19 epidemic in China [24]	*Science*
9	10	2017	Anticipating the emergence of infectious diseases [21]	*Journal of The Royal Society Interface*
10	10	2018	Wastewater-based epidemiology biomarkers: Past, present and Future [25]	*Trends in Analytical Chemistry*

## 4. Discussion

### 4.1. General Information

In this study, we perform a systematic bibliometric analysis of infectious disease early warning research using Web of Science Core Collection publications from 1999 to 2024. The analysis includes 789 English-language articles published in 387 journals, containing 1029 co-cited references from 3300 institutions across 344 countries/regions.

Our results demonstrate a consistent upward trend in publications related to infectious disease early warning systems, reflecting growing scholarly attention to this field. Publication output peaked in 2021, likely in response to the COVID-19 pandemic. The COVID-19 pandemic has served as a wake-up call, dramatically elevating global awareness of the indispensable role that robust early warning systems play in pandemic preparedness and response. The insights gained from COVID-19 triggered a paradigm shift in global health security thinking, making early warning a central pillar of pandemic prevention strategies. Given the global impact of COVID-19 and other EIDs/Re-EIDs, research in disease surveillance and early warning has advanced significantly worldwide. Notably, publication volumes have remained at elevated levels through 2024, suggesting that the pandemic has effected an enduring transformation rather than a temporary reaction in global surveillance paradigms.

### 4.2. Research Hotspots and Frontiers

#### 4.2.1. Climate, Machine Learning, and Avian Influenza

As keywords encapsulate the core essence of the literature, analyzing keyword co-occurrence frequency and centrality can facilitate the identification of research hotspots [26]. We found that “climate change”, “outbreak”, and “public health” were the only three keywords which appeared in both the top 10 keywords of highest frequency and centrality, except for conceptual terms such as “infectious disease” and “disease”, suggesting that these keywords are the focus of current research.

It is widely recognized that climate and other environmental changes directly affect infectious disease patterns [27], and disease agents such as viruses, bacteria, and related vectors to diseases are sensitive to temperature and humidity [28]. The COVID-19 pandemic provided striking examples of this complex interplay, where a number of countries suffered from natural hazard events during the COVID-19 pandemic [29,30]. Quigley MC et al. [31] suggest that there is an increased risk of compounding impacts originating from meteorological and geophysical hazards during the pandemic, including both the effects of the natural disaster being worse and the additional spread of infectious disease. These observations have formed a key consensus in the scientific community: the interaction between climate and health requires comprehensive monitoring, and traditional single-hazard early warning systems are insufficient to address compound risks. As a result, there is broad consensus that integrated early warning systems, with special emphasis on climate change and public health, play an essential role in reducing compound and cascading impacts [32].

Furthermore, according to keyword cluster analysis, “machine learning”, “Avian influenza”, and “Clinical manifestations” were also regarded as the current research hotspots. The availability of large-scale and diverse datasets, coupled with advances in computational infrastructure, has prompted scholars to explore machine learning techniques to improve infectious disease surveillance and early warning capabilities [33,34]. Many investigations have leveraged diverse machine learning methodologies, such as random forests, decision tree models, and deep neural network architectures to analyze various datasets. These include electronic health records, genomic sequencing data, and social media metadata, with the aim of forecasting the spatiotemporal dynamics and emergence of infectious diseases [35,36]. For instance, Raina MacIntyre C et al. [37] propose the use of artificial intelligence as well as machine learning to analyze massive open-source datasets from news platforms, social media, and other digital sources, thereby generating actionable early warning signals for emerging epidemics. Chiu HR et al. [38] employed machine learning to analyze hospital admissions for influenza-like illness, successfully modeling severe illness or mortality risk. This demonstrates the utility of machine learning in addressing the complex challenges posed by emerging infectious diseases. Most of the current research on avian influenza focuses on H5N1, H7N9, or H10N8, which have caused human infections in multiple Asian and European nations [39]. The ongoing prevalence of avian viruses, particularly H5N1, within avian populations, coupled with their heightened potential for cross-species transmission to humans and other mammals, may present an unprecedented opportunity to prepare for the next pandemic threat. This opportunity lies in addressing key scientific questions such as viral cross-species transmission mechanisms and host adaptive mutations [40,41]. Thus, the core objective of multidimensional research on avian influenza by researchers is to enhance the predictive capabilities and intervention efficiency of global public health emergency responses through prospective studies.

Keyword burst analysis encompasses two fundamental parameters: the intensity of the burst and its temporal persistence. A longer-lasting burst typically correlates with higher intensity, indicating the sustained influence of a research topic during a specific period. Keywords that maintain continuous relevance from their initial emergence in a given year are often identified as persistent hotspots and as indicative of ongoing research frontiers [42]. As presented in the keywords burst map, “influenza” and “climate change” saw the most extended burst periods, indicating that scholars continue to pay attention to the outbreak of influenza and the association between climate change and early warning of infectious diseases. In addition, “sars” ranked second in the duration of citation burst, suggesting a large amount of research was attracted to investigate monitoring and early warning systems to prevent and control SARS. The keyword “virus” had the second highest burst intensity. In 2003, the WHO issued a worldwide alert for an unknown emerging illness, later named SARS. Since then, we have witnessed the emergence of several influenza viruses, including avian influenza H5N1, H7N9, and H10N8, and variant influenza A H3N2 virus, which seriously endangers human health [43]. In response, various surveillance systems have been developed to monitor the emergence of infectious diseases; researchers have also attached great importance to the study of viruses and published relevant articles.

#### 4.2.2. Wastewater-Based Epidemiology, One Health, and Artificial Intelligence

Recently, “Wastewater-Based Epidemiology”, “sewage”, “One Health”, and “artificial intelligence” have become the present research trends. Wastewater-based epidemiology was first proposed by researchers from the US Environmental Protection Agency in 2001, who hypothesized that the analysis of drug residues in sewage could be linked to population usage [44]. Thereafter, sewage epidemiology is widely used in tracking illicit drug consumption in various countries [45,46]. At present, WBE has successfully assessed consumption and trends in the use of alcohol, tobacco, and caffeine through the analysis of sewage [47,48]. Water-borne viruses are a kind of microbial community widely existing in sewage, and they are also the main infection and diffusion mode of various human diseases [49]. WBE operates on the premise that analyzing aggregated wastewater from populations enables the comprehensive real-time monitoring of infectious disease spread, antimicrobial resistance dynamics, and the emergence of novel outbreaks at the community level. Therefore, researchers have proposed the use of WBE for the detection of water-borne diseases, such as poliovirus, hepatitis virus, and norovirus, and it has been applied in Japan and Israel [50]. In addition, some animal infectious viruses, such as avian influenza and SARS virus, are often detected in sewage, which is the basis for scholars to propose the application of WBE to warn SARS-CoV-2 virus [51].

The outbreak of the COVID-19 pandemic in 2020 has revitalized scientific attention toward WBE, demonstrating its dual utility as both a predictive indicator and sustainable monitoring mechanism for tracking community-level pathogen transmission dynamics. WBS remains a cost-effective and non-invasive methodology for the large-scale long-term monitoring of SARS-CoV-2 prevalence within populations. [52]. Recent studies employing WBE for routine monitoring of human adenoviruses and enteroviruses have revealed distinct seasonal patterns, with viral concentrations peaking consistently during the spring and summer months [53]. From a public health perspective, these findings carry significant implications for the predictive surveillance of potential outbreak waves and the timely implementation of preventive measures. Importantly, studies demonstrate WBE’s particular utility as a complementary surveillance tool when clinical monitoring systems are inadequate or unavailable [54,55]. During endemic phases, WBE’s objectives and potential evolve from early case identification to delivering situational assessments of viral infection trends within populations, offering actionable insights for public health strategy adjustment. These results align with growing evidence that environmental surveillance can effectively bridge gaps in traditional health monitoring systems, especially for pathogens with high rates of asymptomatic transmission. The methodologies devised during the SARS-CoV-2 pandemic and their analytical frameworks hold significant utility for future wastewater surveillance initiatives. Such approaches can facilitate the discovery of emerging zoonotic pathogens and enable the early detection of potential pandemic threats, thereby enhancing global preparedness for infectious disease outbreaks [56]. The findings underscore the critical need for public health systems to strengthen real-time surveillance infrastructure by integrating wastewater-based epidemiology (WBE) as a core component of disease monitoring frameworks and to allocate funding to expand WBE monitoring networks, particularly in resource-limited settings where clinical surveillance is sparse. By institutionalizing WBE, policymakers can transform reactive outbreak responses into proactive, data-driven public health strategies.

More than 30 new human pathogens have been detected over the past three decades, 75 percent of which have originated in animals [57], which confirms the view that the majority of emerging infectious diseases originate in animals, both wild and domestic [58]. This highlights the critical need to strengthen surveillance systems at the human–animal–environment interface and improve early warning systems for zoonotic diseases, which provides compelling justification for implementing the One Health approach. The term “One Health” was first used in 2003–2004; the emergence of SARS put a spotlight on the need for a more integrated global framework for improved global disease prevention, surveillance, control, and, in particular, for the inclusion of wildlife health. It is officially defined by the WHO as the “integrated, unifying approach that aims to sustainably balance and optimize the health of people, animals, and ecosystems” [59]. With the development of early warning for infectious disease detection, the One Health paradigm has emerged as a transformative framework for anticipating outbreaks through early warning systems, emphasizing the interdependencies among human, animal, and environmental health [60]. Effective One Health infectious disease surveillance systems require collaboration among human, animal, and environmental health agencies to establish communication networks and multi-level coordination systems, ensuring the integration of data across all health domains [61].

COVID-19 has dramatically elevated One Health approaches to becoming priorities in global health and development agendas [62]. The COVID-19 pandemic has demonstrated that traditional single-disciplinary prevention and control models have struggled to achieve effective outcomes when confronting complex transmission networks of pathogens like SARS-CoV-2 across human–animal–ecosystem interfaces. Recognizing the importance of the One Health approach in detecting and controlling future pandemics, more countries are prioritizing and practicing the prevention of zoonotic disease outbreaks and the promotion of interdisciplinary collaboration [63,64]. Genomic sequencing technologies have enabled cross-species pathogen tracking, such as monitoring H5N1 avian influenza variant strains [65,66]. The integration of environmental DNA detection with AI-driven predictive models has facilitated the real-time dynamic assessment of wildlife pathogen reservoirs [67,68]. These technological breakthroughs provide essential tool-based support for establishing risk monitoring within the One Health framework. For example, Thailand’s federal government initiated a pilot avian influenza surveillance initiative, engaging multi-sectoral stakeholders from human health, veterinary, and environmental domains to monitor influenza A viruses across human populations, waterfowl, and poultry [69]. This innovative pilot framework establishes a strategic model for designing, integrating, and strengthening One Health surveillance methodologies. By fostering cross-disciplinary collaboration, such systems aim to address emerging global pathogen risks proactively and enhance collective health security measures, providing a replicable template for multi-jurisdictional disease monitoring. The post-COVID era demands a paradigm shift from reactive outbreak response to proactive One Health-driven prevention. By integrating human, animal, and environmental surveillance, policymakers can detect threats earlier, healthcare systems can prepare more effectively, and global health security can be strengthened.

In recent years, with the advancement of artificial intelligence, this technology has been widely applied in the realm of infectious disease prevention and control [70]. Artificial intelligence has rapidly emerged as a cutting-edge frontier in infectious disease prevention and control, primarily due to its superior computational capacity for analyzing complex datasets and enabling real-time decision-making [71]. Conventional statistical approaches have proven inadequate for processing multifaceted data streams, including genomic sequences, environmental surveillance metrics, and human mobility patterns [72]. Advanced deep learning architectures in AI systems demonstrate unique capabilities to decode such complex high-dimensional data, significantly enhancing epidemic prediction accuracy [73,74]. Furthermore, the integration of AI into intelligent healthcare systems has facilitated its comprehensive application across the entire clinical workflow, from diagnosis to treatment optimization [75]. The COVID-19 pandemic demonstrated that rapid, accurate pathogen detection constitutes a critical determinant of effective epidemic control [76]. Contemporary integration of artificial intelligence with cutting-edge biotechnologies—including synthetic biology platforms, transcriptomic profiling, high-throughput mass spectrometry, and advanced imaging modalities—has significantly enhanced both diagnostic precision and antimicrobial resistance prediction capabilities [77]. In addition to assisting in diagnosis, AI systems are also being deployed across multiple dimensions of epidemic management, including outbreak early warning systems, contact tracing, infection diagnostics, drug discovery, and pharmaceutical design [78]. Contact tracing is the process of tracing potential routes of transmission of an infection in a population, with the aim of isolating people who may have been exposed and reducing further transmission, and has been used for several diseases such as tuberculosis and Ebola [79]. Harnessing artificial intelligence models enables the identification of novel behavioral patterns to enhance social distancing compliance monitoring, improve infection risk assessment accuracy, and optimize COVID-19 transmission mitigation strategies [80,81]. Leading pharmaceutical companies are leveraging AI to accelerate vaccine development pipelines. Moderna, a pioneer in mRNA COVID-19 vaccines, has implemented AI-driven robotic platforms capable of generating over 1000 mRNA sequences monthly [82].

The COVID-19 pandemic has unequivocally demonstrated the critical role of artificial intelligence in epidemiological early warning systems. AI technologies provide three fundamental advantages: earlier outbreak detection through predictive modeling prior to laboratory confirmation, more precise intervention via data-driven containment strategies, and improved healthcare efficiency by automating surveillance tasks to reduce clinician workload. The integration of AI into global health infrastructure facilitates the creation of proactive intelligence-based infectious disease warning systems. As an essential element of future pandemic preparedness, AI’s comprehensive contributions to both prevention and response highlight its vital role in protecting global public health. It is worth noting that the digital surveillance of infectious diseases may undermine the fundamental rights of individuals, especially with regard to privacy protection and data confidentiality [83]. To balance public health benefits with individual rights, the deployment of AI in infectious disease monitoring must be guided by integrated medical standards, ethical principles, and legal regulations [84].

### 4.3. Advancing Infectious Disease Early Warning: Implementing the Human–Animal-Environment Monitoring System

Our bibliometric analysis reveals wastewater-based epidemiology and AI to be emergent research frontiers through the timeline view of keywords (Figure 7), keyword burst analysis (Figure 8), and top 10 cited references (Table 3).

WBE’s core principle lies in the fact that human excreta and domestic sewage contain pathogens or biomarkers, which can be quantitatively detected using technologies such as RT-qPCR and metagenomic sequencing, thereby inferring the epidemic trends of infectious diseases within communities [85]. The top-cited references [14,17] demonstrate WBE’s utility in community-level SARS-CoV-2 surveillance, validated by its cost-effectiveness. Compared to traditional monitoring methods, WBE has significant advantages, as it does not rely on individual proactive medical treatment or testing, especially suitable for scenarios with a high proportion of asymptomatic infected individuals, such as COVID-19 and norovirus, effectively filling gaps in clinical data [86]. For instance, as mentioned earlier, Japan and Israel have applied WBE to monitor poliovirus and hepatitis viruses. Additionally, single wastewater detection costs are relatively low, making it suitable for large-scale and long-term monitoring [16]. The timeline (Figure 7) shows WBE’s rise post-2020, reflecting its adoption for real-time pathogen tracking.

AI-driven predictive models leverage algorithms such as machine learning and deep learning, utilizing big data to achieve the dynamic simulation and prediction of infectious disease transmission [87]. Keywords such as “machine learning” (Cluster #4) and “artificial intelligence” (Figure 8) highlight AI’s integration into predictive modeling. Infectious disease transmission involves data such as climate and human mobility, which are difficult to handle with traditional statistical methods. The cited studies emphasize AI’s capacity to analyze complex datasets for early outbreak detection. As mentioned earlier, AI can detect outbreaks earlier by using modeling, enable more precise interventions, and improve medical efficiency through automated monitoring, significantly reducing the human burden on public health. Integrating AI into global health infrastructure helps create intelligence-based infectious disease early warning systems.

Over the past three decades, the frequency of emerging and re-emerging infectious diseases has increased globally, with escalated threats from infectious diseases and heightened transmission risks due to globalization [88]. Traditional surveillance systems struggle to capture signals in the early stages of cross-species pathogen transmission, whereas real-time monitoring infrastructure, such as WBE networks and AI-driven early warning models, can detect complex systemic risks [89]. For example, WBE can simultaneously monitor zoonotic viruses, such as avian influenza viruses, providing data support for the “One Health” framework [90]. Governments’ prioritization of investments in real-time monitoring infrastructure essentially embodies the public health philosophy of “prevention as the priority”. As the COVID-19 pandemic has revealed the fragility of global health, only by prioritizing investments in real-time monitoring infrastructure can we take the initiative in future infectious disease challenges and safeguard population health and socioeconomic stability.

However, in the face of compound risks such as the cross-species transmission of pathogens and ecological environmental changes, single technical measures are difficult to fully cover monitoring needs, and the above technologies need to be embedded in a more holistic public health governance framework. Within the “One Health” framework, implementing a “human–animal–environment monitoring system” to strengthen infectious disease early warning requires establishing a cross-dimensional collaborative monitoring network.

Firstly, for human monitoring, active detection should be enhanced by setting up infectious disease surveillance sentinel sites in medical institutions such as sentinel hospitals and communities. These sites should use electronic medical record systems to capture and analyze case data such as influenza-like illnesses. Wastewater epidemiology can be employed to detect pathogen nucleic acids in sewage, assessing population infection trends, particularly for asymptomatic cases. Secondly, for animal monitoring, efforts should focus on both wild animals and domestic livestock/poultry. Monitoring stations should be established in high-risk zones for zoonotic diseases to detect pathogens such as avian influenza viruses through fecal and blood samples, with special attention to viral strains with high cross-species transmission risks. Regular sampling and testing of large-scale farms can help with the early warning of animal-borne disease outbreaks. Thirdly, for environmental monitoring, data on temperature, humidity, precipitation, and air pollution should be collected to analyze their correlations with pathogen survival and vector breeding cycles.

Furthermore, a unified data management platform must be developed to integrate human clinical data, animal disease reports, and environmental monitoring data, ensuring standardized and real-time data sharing. Departments such as health, agriculture, and ecology should jointly formulate unified monitoring standards, data-sharing protocols, and emergency response procedures, clarifying the roles of different stakeholders. Artificial intelligence, such as deep learning algorithms, can analyze historical data to build predictive models of infectious disease transmission risks, enabling early warning. The core of the “human–animal–environment monitoring system” lies in treating human health, animal health, and ecosystem health as an inseparable whole. Through cross-dimensional data integration and collaborative mechanisms, it achieves the early identification and warning of infectious diseases, safeguarding public health security.

### 4.4. Limitations and Future Research Perspectives

Our study has several significant limitations that warrant careful consideration when interpreting the results. On the one hand, the exclusive reliance on English-language articles within the Web of Science core database may have led to the omission of valuable research published in other languages. This is particularly relevant given our finding that China ranked second in research productivity; studies published in Chinese could potentially alter our conclusions. On the other hand, by focusing solely on journal articles, we excluded important gray literature sources, including WHO technical reports, government surveillance data, and conference proceedings. These documents often contain timely and policy-relevant insights that could influence the understanding of the research field.

To mitigate these limitations in future investigations, we propose three strategies: (1) broadening database searches to incorporate regional repositories such as CNKI; (2) leveraging machine translation tools to include non-English publications; and (3) systematically retrieving gray literature through platforms like Google Scholar and organizational databases.

## 5. Conclusions

This bibliometric analysis provides an updated, global, and contextualized perspective of the infectious disease early warning system as of September 2024. The number of articles published peaked in 2021, and the importance of early warning systems for infectious disease has been highlighted due to COVID-19. Research related to the role of early warning systems in detecting and responding to infectious disease outbreaks over the past two decades focused on climate change, influenza, SARS, virus, machine learning, warning signals and systems, artificial intelligence, and so on. Current research hotspots include wastewater-based epidemiology, sewage, One Health, and artificial intelligence, as well as the early warning and monitoring of COVID-19. In conclusion, research foci in this area have evolved from focusing on climate–disease interactions to pathogen monitoring systems and ultimately to the “One Health” integrated framework. Our research findings underscore the imperative for public health policymakers to prioritize investments in real-time surveillance infrastructure, particularly wastewater-based epidemiology and AI-driven predictive models, and strengthen interdisciplinary collaboration frameworks under the One Health paradigm. Developing an integrated human–animal–environment monitoring system will serve as a critical development direction for early warning systems for epidemics.

## Figures and Tables

**Table 1:** Top 10 institutions in terms of highest centrality.

Rank	Centrality	Publications	Institution	Country
1	0.22	17	World Health Organization	International organization
2	0.20	18	Harvard University	United States
3	0.14	36	Chinese Center for Disease Control & Prevention	China
4	0.12	21	University of California System	United States
5	0.09	22	Chinese Academy of Sciences	China
6	0.06	9	University System of Maryland	United States
7	0.06	9	Centre National de la Recherche Scientifique	France
8	0.05	21	University of London	United Kingdom
9	0.05	6	Universite Paris Cite	France
10	0.04	16	London School of Hygiene & Tropical Medicine	United Kingdom

**Table 2:** Top ten keywords of highest frequency and centrality.

Rank	Frequency	Centrality	Keyword	Centrality	Frequency	Keyword
1	181	0.31	infectious diseases	0.31	181	infectious diseases
2	64	0.06	transmission	0.16	50	disease
3	57	0.06	virus	0.15	53	climate change
4	53	0.10	outbreak	0.11	34	early warning system
5	53	0.15	climate change	0.1	53	outbreak
6	50	0.16	disease	0.1	21	children
7	48	0.07	public health	0.07	48	public health
8	48	0.04	surveillance	0.07	36	early warning
9	39	0.04	infection	0.07	31	risk
10	38	0.05	epidemiology	0.07	25	emerging infectious diseases

## Data Availability

Data will be available from the corresponding author (Hui Lu) on request.

## Abbreviations

The following abbreviations are used in this manuscript:

EIDs	emerging infectious diseases
Re-EIDs	re-emerging infectious diseases
WHO	World Health Organization
WoSCC	Web of Science Core Collection
WBE	wastewater-based epidemiology
AI	artificial intelligence
